# Association of the PECAM-1 (Leu125Val) and P-Selectin (Thr715Pro) Gene Polymorphisms With Unexplained Spontaneous Miscarriages

**DOI:** 10.7759/cureus.21859

**Published:** 2022-02-03

**Authors:** Nikolaos Vlachadis, Vassilios Tsamadias, Maria Siori, Nikolaos Vrachnis, Emmanuel Economou

**Affiliations:** 1 Clinical Laboratory for Therapeutic Individualization, National and Kapodistrian University of Athens Medical School, Aretaieio Hospital, Athens, GRC; 2 General Medicine, National Health Services, Athens, GRC; 3 Third Department of Obstetrics and Gynecology, National and Kapodistrian University of Athens Medical School, Attiko Hospital, Athens, GRC

**Keywords:** p-selectin, pecam-1, polymorphism, spontaneous abortion, pregnancy, miscarriage

## Abstract

Introduction: The aim of this study was to investigate the possible effect of the PECAM-1-C373G (Leu125Val) and P-Selectin-A37674C (Thr715Pro) polymorphisms in unexplained spontaneous abortions.

Methods: In a case-control design, Greek nulligravida women with recurrent idiopathic miscarriages <20 weeks of gestation and fertile controls were genotyped by pyrosequencing.

Results: There was no significant association of the PECAM-1-C373G (Leu125Val) polymorphism with recurrent abortions. Although the P-Selectin-A37674C (Thr715Pro) polymorphism was not associated with miscarriages overall, the association was statistically significant for younger women (carriers of the P-Selectin-37674C allele: <35 years: odds ratio (OR) = 3, 95% confidence interval (CI): 1.13-7.97, p = 0.023; <30 years: OR = 6.75, 95%CI: 2.02-22.58, p = 0.002). In comparison with CC/AA genotype, the combined carriers of the PECAM-1-373G and P-Selectin-37674C alleles had OR =8.81 (95%CI: 1.07-72.50, p = 0.024). The association of the coexistence of the two polymorphisms was stronger in younger women (<35 years: OR = 12.07, 95%CI: 1.38-105.68, p = 0.014; <30 years: OR = 65, 95%CI: 3.38-1251.28, p = 0.001), and late (OR = 10.64, 95%CI: 1.16-97.60, p = 0.024) and second-trimester miscarriages (OR = 26, 95%CI: 1.84-367.71, p = 0.014). The association between carriage of the P-Selectin-37674C allele and recurrent miscarriages was significant for younger women.

Conclusion: The coexistence of the PECAM-1-373G and P-Selectin-37674C alleles increased the miscarriage risk for the total population studied, suggesting an interaction between the two polymorphisms, more pronouncedly in younger women and the association was stronger for late fetal loss.

## Introduction

Miscarriage is the most common major complication of pregnancy, and it affects 15-25% of clinically recognized pregnancies. Almost 5% of pregnant women suffer recurrent pregnancy loss, defined by two or more failed pregnancies at <20 weeks of gestation. This is a multifactorial disorder, and various etiologic factors have been proposed including cytogenetic abnormalities, immunologic disorders, anatomic, endocrine and metabolic defects, and infectious agents, albeit in approximately 50% of the cases the causes remain unknown [1]. The potential association of a great number of single-nucleotide polymorphisms (SNPs) with unexplained spontaneous miscarriages has been studied intensively, mostly with contradictory results [2].

Platelet endothelial cell adhesion molecule (PECAM-1) and P-Selectin are cell adhesion molecules expressed on the membrane of platelets, leukocytes, and endothelial cells. These two receptors exert pleiotropic effects in inflammation, platelet function, and thrombosis, as well as angiogenesis [3,4]. Two SNPs, PECAM-1-C373G (Leu125Val) (rs281865545) and P-Selectin-A37674C (Thr715Pro) (rs6136), have been implicated in vascular thrombosis [5,6]. Implantation and placentation are complex procedures including regulation of blood coagulation and inflammatory and angiogenic phenomena [7,8]. Given the important role of PECAM-1 and P-Selectin in these processes, the above-mentioned two SNPs are promising candidates for identification of association with spontaneous abortions.

The possible effect of these two polymorphisms in pregnancy losses has not been investigated in the literature. Recently, we examined their influence on implantation after in vitro fertilization and reported that the coexistence of PECAM-1-373G and P-Selectin-37674C alleles significantly increased the risk of implantation failures, especially among younger women [9]. In this study we aim to discuss the role of the PECAM-1-C373G (Leu125Val) and P-Selectin-A37674C (Thr715Pro) polymorphisms and their interaction as risk factors for spontaneous miscarriages.

## Materials and methods

This was a case-control study set up in Aretaieio University Hospital, Athens, Greece. The study was in compliance with the Declaration of Helsinki on medical protocol and ethics. The Ethics Committee of our institution approved the study protocol and written informed consent was obtained from all the participants.

The study group comprised nulligravida women who had experienced spontaneous miscarriages before completion of 20 weeks of gestation, with the same partner. For each patient, a thorough medical and obstetric history was obtained. All cases were referred to our department after primary evaluation according to a standard protocol set up to detect known or putative causes of pregnancy loss, consisting of karyotypes in both parents, male partner spermiogram, ultrasonography examination, hysterosalpingogram and/or hysteroscopy, immunologic and infections tests, and comprehensive determination of hormonal status. One hundred and sixty-two unrelated patients negative after the aforementioned primary evaluation were defined as having unexplained recurrent miscarriages and were included in the study. In all pregnancies that ended in miscarriages, the women had been offered an ultrasound scan at around six gestational weeks to confirm viable single intrauterine pregnancy and gestational age. Additionally, in all fetal losses that occurred in the second trimester, pregnancies had been considered low risk after combined non-invasive first-trimester ultrasound and biochemical screening. All patients had suffered their last pregnancy loss during the previous six months and the age of each participant was considered to be that at the time of last miscarriage.

The control population consisted of 60 unrelated women aged >40 years who had completed their childbearing with at least one living child born at term, and no personal history of miscarriages, infertility, pregnancy complications or thrombosis, and no family history of vascular events at age <65 years. All cases and control subjects were of Greek origin, healthy, and did not receive any medications during the study period.

Blood samples were collected from all participants and genomic DNA was extracted from the blood collected, after which polymerase chain reaction (PCR) amplification genotyping was performed by pyrosequencing, as previously described [9]. The Hardy-Weinberg equilibrium (HWE) was tested by comparing the observed genotype frequencies with those expected under HWE with the chi-square goodness-of-fit test using HPlus 2.5 software. Quantitative variables were expressed as mean ± standard deviation (SD) and/or median ± interquartile range (IQR), and categorical variables were presented as numbers and percentages (%). Genotype frequencies were compared using Pearson’s chi square and Fisher's exact tests as appropriate and the strength of the associations was expressed as odds ratio (OR) with corresponding 95% confidence interval (95%CI). OR trends were evaluated using the chi-square test for trend. p-Value <0.05 was considered statistically significant. Statistical analysis was performed using MedCalc® Statistical Software version 12.7.7 (2013 MedCalc Software bvba, Ostend, Belgium). All the genetic associations were determined separately for the younger and the older age groups defined as women aged <35 and ≥35 years (younger and older half of our case population) as well as those aged <30 years. A secondary analysis was also performed according to the gestational age at miscarriage. Specifically, women who had miscarriages only at <10 weeks of gestation (embryonic period) were classified as “early miscarriages,” whereas those with at least one miscarriage at ≥10 weeks (fetal period) were classified as “late miscarriages.” Furthermore, participants with at least one pregnancy loss at >12 weeks (second-trimester miscarriages) were separately evaluated.

## Results

A total of 162 women with primary recurrent spontaneous miscarriages and 60 control subjects were included in this study. The women in the study group ranged in age from 23 to 40 years (mean: 33.8 ± 3.9 years, median: 34 years, IQR: 31-37 years). The mean number of miscarriages in the group of patients was 2.6 ± 0.8. The mean number of term pregnancies that resulted in live births in the control subjects was 2.1 ± 0.5. Among the cases, 104 women (64.2%) had exclusively miscarriages before the completion of 10 gestational weeks (early miscarriages) and 58 women (35.8%) had at least one miscarriage after 10 weeks of gestation (late miscarriages), whereas in 19 women (11.7%) at least one miscarriage occurred after 12 weeks of gestation (second-trimester miscarriages).

Genotype frequency distributions of both studied variants were in HWE in cases and control groups (PECAM-1-C373G: cases: p = 0.881, controls: p = 0.449, P-Selectin-A37674C: cases: 0.929, controls: 0.684).

The frequencies of PECAM-1-373G and P-Selectin-37674C allele carriers are presented in Table 1. There was no statistically significant association between carriership of the PECAM-1-373G allele and miscarriage risk (OR = 1.19, 95%CI: 0.62-2.28, p = 0.610). Overall, the frequency of P-Selectin-37674C allele carriers was higher among women with spontaneous miscarriages, than in the control subjects (20.4% vs. 10%) but the difference was marginally non-significant (OR = 2.30, 95%CI: 0.91-5.81, p = 0.071). However, there was a statistically significant association between possession of the combination of the PECAM-1-373G and P-Selectin-37674C and risk of fetal loss. In comparison with double wild-type homozygotes (genotype: CC/AA), the combined carriers of the PECAM-1-373G and P-Selectin-37674C alleles had OR =8.81 (95%CI: 1.07-72.50, p = 0.024).

**Table 1:** Distribution of PECAM-1-C373G (Leu125Val) and P-Selectin-A37674C (Thr715Pro) polymorphisms in cases and control groups and risk associations of polymorphic allele carriers with spontaneous miscarriages. OR, odds ratio; CI, confidence interval.

Genotypes	Cases		Controls		OR	95%CI	p-Value
	Ν (=162)	(%)	Ν (=60)	(%)			
PECAM-1-C373G (Leu125Val)							
CC	43	26.5	18	30	1.19	0.62-2.28	0.610
PECAM-1-373G carriers	119	73.5	42	70			
P-Selectin-A37674C (Thr715Pro)							
AΑ	129	79.6	54	90	2.30	0.91-5.81	0.071
P-Selectin-37674C carriers	33	20.4	6	10			
PECAM-1-C373G (Leu125Val) and P-Selectin-A37674C (Thr715Pro) combination							
CC/AA	31	19.1	13	21.7	8.81	1.07-72.50	0.024
Carriers of combination of PECAM-1-373G and P-Selectin-37674C	21	13.0	1	1.7			

The distribution of the two gene variants was further analyzed according to age (Table 2). The association of the PECAM-1-C373G (Leu125Val) genetic heterogeneity remained non-significant in both the younger (aged <35 years) and older (aged ≥35 years) groups of our population. The prevalence of the PECAM-1-373G allele showed a slight increase toward younger ages (≥35 years: 70.5%, <35 years: 76.2%) but the trend was statistically insignificant (p for trend = 0.341). However, a statistically significantly increasing trend of P-Selectin-37674C allele carriers’ prevalence toward younger ages was detected (p for trend < 0.001). The P-Selectin-37674C allele was detected in 25% of patients aged <35 years, which was significantly higher than the proportion of 10% among controls (OR = 3, 95%CI: 1.13-7.97, p = 0.023), whereas the highest risk was observed for the youngest subgroup (aged <30 years) (OR = 6.75, 95%CI: 2.02-22.58, p = 0.002). The combination of PECAM-1-373G and P-Selectin-37674C alleles had OR =12.07 (95%CI: 1.38-105.68, p = 0.014) for subjects aged <35 years, whereas the association was not statistically significant for older women (OR = 6.12, 95%CI: 0.68-55.25, p = 0.119) (p for trend < 0.001). The relative risk for combined carriers was further increased (OR = 65, 95%CI: 3.38-1251.28, p=0.001) for the youngest subgroup of participants (aged <30 years).

**Table 2:** Distribution of PECAM-1-C373G (Leu125Val) and P-Selectin-A37674C (Thr715Pro) polymorphisms in cases and control groups and risk associations of polymorphic allele carriers with spontaneous miscarriages stratified by age. OR, odds ratio; CI, confidence interval.

			Cases (N = 162)				Controls (N = 60)						
Polymorphisms	Age (years)		Polymorphic allele carriers		Wild-type homozygotes		Polymorphic allele carriers		Wild-type homozygotes		OR	95%CI	p- Value
		N	Ν	(%)	Ν	(%)	Ν	(%)	Ν	(%)			
PECAM-1-C373G (Leu125Val)	≥35	78	55	70.5	23	29.5	42	70	18	30	1.02	0.49-2.14	0.948
	Total	162	119	73.5	43	26.5					1.19	0.62-2.28	0.610
	<35	84	64	76.2	20	23.8					1.37	0.65-2.89	0.406
	<30	21	16	76.2	5	23.8					1.37	0.44-4.31	0.590
P-Selectin-A37674C (Thr715Pro)	≥35	78	12	15.4	66	84.6	6	10	54	90	1.64	0.58-4.65	0.351
	Total	162	33	20.4	129	79.6					2.30	0.91-5.81	0.071
	<35	84	21	25	63	75					3	1.13-7.97	0.023
	<30	21	9	42.9	12	57.1					6.75	2.02-22.58	0.002
PECAM-1-C373G (Leu125Val) and P-Selectin-A37674C (Thr715Pro) combination	≥35	78	8	10.3	17	21.8	1	1.7	13	21.7	6.12	0.68-55.25	0.119
	Total	162	21	13.0	31	19.1					8.81	1.07-72.50	0.024
	<35	84	13	15.5	14	16.7					12.07	1.38-105.68	0.014
	<30	21	5	23.8	1	4.8					65	3.38-1251.28	0.001

The distribution of the two gene variants was further analyzed by subgrouping cases based on the gestational period of miscarriages (Table 3). There was no significant association of the PECAM-1-C373G (Leu125Val) polymorphism with embryonic (<10 weeks) or late miscarriages (≥10 weeks). The prevalence of the PECAM-1-373G allele displayed a non-significant increasing trend toward later miscarriages (<10 weeks: 71.2%, ≥10 weeks: 77.6%, p for trend = 0.319). The prevalence of the PECAM-1-373G allele rose further among women with second-trimester miscarriages (>12 weeks: 89.5%) but the effect was again non-significant (OR = 3.64, 95%CI: 0.76-17.44, p = 0.131). Similarly, the distribution of the P-Selectin-37674C allele showed no significant trend for gestational age of miscarriages (<10 weeks: 21.2%, ≥10 weeks: 19.0%, p for trend = 0.094). Combined carriers of the two polymorphic alleles had an increased risk of fetal miscarriages (≥10 weeks) (OR = 10.64, 95%CI: 1.16-97.60, p = 0.024), which was notably elevated for miscarriages >12 gestational weeks (OR = 26, 95%CI: 1.84-367.71, p = 0.014).

**Table 3:** Distribution of PECAM-1-C373G (Leu125Val) and P-Selectin-A37674C (Thr715Pro) polymorphisms in cases and control groups and risk associations of polymorphic allele carriers with spontaneous miscarriages stratified by gestational age. OR, odds ratio; CI, confidence interval.

			Cases (N = 162)				Controls (N = 60)							
Polymorphisms	Gestational age (weeks)		Polymorphic allele carriers		Wild-type homozygotes		Polymorphic allele carriers		Wild-type homozygotes			OR	95%CI	p- Value
		N	Ν	(%)	Ν	(%)	Ν	(%)	Ν		(%)			
PECAM-1-C373G (Leu125Val)	<10	104	74	71.2	30	28.8	42	70	18	30		1.06	0.53-2.12	0.888
	Total	162	119	73.5	43	26.5						1.19	0.62-2.28	0.610
	≥10	58	45	77.6	13	22.4						1.48	0.65-3.40	0.348
	>12	19	17	89.5	2	10.5						3.64	0.76-17.44	0.131
P-Selectin-A37674C (Thr715Pro)	<10	104	22	21.2	82	78.8	6	10	54		90	2.41	0.92-6.34	0.068
	Total	162	33	20.4	129	79.6						2.30	0.91-5.81	0.071
	≥10	58	11	19.0	47	81.0						2.11	0.72-6.13	0.166
	>12	19	4	21.1	15	78.9						2.40	0.60-9.62	0.242
PECAM-1-C373G (Leu125Val) and P-Selectin-A37674C (Thr715Pro) combination	<10	104	12	11.5	20	19.2	1	1.7	13		21.7	7.80	0.90-67.38	0.072
	Total	162	21	13.0	31	19.1						8.81	1.07-72.50	0.024
	≥10	58	9	15.5	11	19.0						10.64	1.16-97.60	0.024
	>12	19	4	21.1	2	10.5						26	1.84-367.71	0.014

## Discussion

To our knowledge, the present pilot study of strictly selected cases with unexplained spontaneous miscarriages and fertile controls was the first to investigate the potential role of the PECAM-1-C373G (Leu125Val) and P-Selectin-A37674C-(Thr715Pro) SNPs in pregnancy loss. Our results demonstrated that the coexistence of the polymorphic alleles (PECAM-1-373G and P-Selectin-37674C) of the two studied polymorphisms is associated with statistically significantly increased risk of spontaneous miscarriages.

In our sample, we detected no association of the PECAM-1-C373G (Leu125Val) polymorphism with the risk of spontaneous abortions overall, although the prevalence of the PECAM-1-373G allele showed increasing but not significant trends toward younger ages and later miscarriages. Possession of the P-Selectin-37674C allele was associated with increased risk of spontaneous abortions, which, while being non-significant overall and across gestational age groups, was found to be significant in younger women. Carriers of the P-Selectin-37674C allele aged <35 years had three-fold higher odds of having miscarriages, with this figure increasing to almost seven-fold in women younger than 30 years of age.

The coexistence of PECAM-1-373G and P-Selectin-37674C alleles was associated with an almost nine-fold increase in miscarriage risk, in comparison with women who did not carry any polymorphic alleles (genotype: CC/AA), illustrating the multiplicative effect on overall risk when the two mutations were combined. The likelihood of miscarriage was increased 12-fold for combined carriers aged <35 years and reached a marked 65-fold increase in combined carriers aged <30 years. Furthermore, possession of the PECAM-1-373G and P-Selectin-37674C combination resulted in OR =10.64 for miscarriages at ≥10 gestational weeks, which increased to OR =26 for second-trimester loss.

Although we failed to detect any significant association between the genetic heterogeneity A37674C of the P-Selectin gene with unexplained miscarriages overall, the P-Selectin-37674C allele was a risk factor for recurrent miscarriages in younger women. Furthermore, the risk of pregnancy loss due to the coexistence of the PECAM-1-373G and the P-Selectin-37674C alleles was also higher among younger women. Increased risks for younger individuals are anticipated since these two SNPs represent inherited risk factors [10]. Moreover, this age-skewed risk profile appears to be the result of the relative impact of the polymorphisms becoming attenuated with advancing age, which is the determinative risk factor for spontaneous miscarriages [11].

The association of the PECAM-1-373G and P-Selectin-37674C alleles combination was stronger with regard to late miscarriages. Inherited thrombophilic factors have been linked to an increased risk of late miscarriages [12]. This finding could be explained either by the lower presence of chromosomal abnormalities - the major confounding factor in miscarriage studies - in late fetal loss resulting in other risk associations becoming significant, or by alterations in the pathophysiology when pregnancy loss occurs at different developmental milestones [13]. Of note, in the present study, an increased prevalence of the combination of the two risk alleles was observed among women who miscarried after a normal first-trimester screening which excluded the majority of chromosomal defects.

The two polymorphisms investigated in this study lead to prothrombotic and proinflammatory phenotypes. There is much accumulated evidence that thrombotic and inflammatory processes play major roles in the pathophysiology of spontaneous abortions [10,12,14,15]. The presence of thrombophilic factors may result in impaired uteroplacental perfusion and abnormal placentation [12]. Increased concentration of inflammatory cells as well as fibrin deposition have been demonstrated at implantation sites, in deciduas, and in placental membranes of women with recurrent pregnancy loss [14]. PECAM-1 and P-Selectin mediate the complex interactions between platelets, leukocytes, and endothelial cells during inflammation and thrombosis [3,4]. PECAM-1 is expressed in human trophoblasts and endometrial cells and appears to be involved in their interactions during implantation and placentation [16,17]. Evidence has also indicated that PECAM-1 is an important mediator in trophoblast invasion and spiral arteries remodeling [18]. Finally, the adhesive interactions mediated by PECAM-1 are involved in angiogenesis, while it has been suggested that they are important in the formation of new vessels [4]. Selectins, including P-Selectin, are well established as the main adhesion molecules at the maternal-fetal interface with crucial roles during the implantation process [19]. Furthermore, P-Selectin receptors participate in the interactions leading to Th1 lymphocyte migration into the decidua which is involved in the rejection of trophoblast cells [20]. It has been suggested that genetic alterations of platelet membrane factors, including PECAM-1 and P-Selectin, are synergistically involved in the pathogenesis of miscarriages [10] and implantation failure [9,21,22]. In a relative study, Dendana et al. [23] found a statistically significant association between the presence of the G allele of another P-Selectin gene polymorphism (P-Selectin-C2123G, rs6127) and recurrent pregnancy loss.

The possible involvement of PECAM-1 and P-Selectin polymorphisms in unexplained miscarriages could be of particular therapeutic interest, given that heparin is capable of binding PECAM-1 and P-Selectin attenuating mediated adhesions and, consequently, reducing the effectiveness of the inflammatory and prothrombotic response [24,25]. If our results are verified by larger studies, carriers of these two SNPs would be most likely to benefit from heparin treatment.

## Conclusions

In conclusion, our pilot data, although limited, suggest that carriership of the P-Selectin-37674C allele enhances miscarriage risk in younger women. Additionally, interaction between the PECAM-1-373G and the P-Selectin-37674C alleles appears to result in a synergistic effect that strongly predisposes to spontaneous miscarriages with stronger associations for late miscarriages and younger women. Future large prospective studies are needed to further clarify the contribution of these polymorphisms in the etiology of pregnancy loss.
